# Are sociodemographic and anthropometric variables effective in screening probable and confirmed sarcopenia in community-dwelling older adults? A cross-sectional study

**DOI:** 10.1590/1516-3180.2022.0141.R1.17082022

**Published:** 2022-11-21

**Authors:** Larissa Franciny de Souza, Laís Coan Fontanela, Amanda Aparecida Oliveira Leopoldino, Vanessa Amaral Mendonça, Ana Lúcia Danielewicz, Ana Cristina Rodrigues Lacerda, Núbia Carelli Pereira de Avelar

**Affiliations:** I PT. Physical Therapist, Department of Health Sciences, Universidade Federal de Santa Catarina (UFSC), Araranguá (SC), Brazil.; II PT. Physical Therapist, Department of Health Sciences, Universidade Federal de Santa Catarina (UFSC), Araranguá (SC), Brazil.; III PT, MSc, PhD. Adjunct Professor, Postgraduate Program, Faculdade de Ciências Médicas de Minas Gerais (FCM-MG), Belo Horizonte (MG), Brazil.; IV PT, MSc, PhD. Associate Professor, Universidade Federal dos Vales do Jequitinhonha e Mucuri (UFVJM), Diamantina (MG), Brazil.; V PT, MSc, PhD. Adjunct Professor, Department of Health Sciences, Universidade Federal de Santa Catarina (UFSC), Araranguá (SC), Brazil.; VI PT, MSc, PhD. Associate Professor, Universidade Federal dos Vales do Jequitinhonha e Mucuri (UFVJM), Diamantina (MG), Brazil.; VII PT, MSc, PhD. Adjunct Professor, Department of Health Sciences, Universidade Federal de Santa Catarina (UFSC), Araranguá (SC), Brazil.

**Keywords:** Sarcopenia, Anthropometry, Early diagnosis, Aging, Body weights and measures, Early detection of disease, Senescence

## Abstract

**BACKGROUND:**

Screening for probable and confirmed sarcopenia using sociodemographic and anthropometric indicators can be a practical, cheap, and effective strategy to identify and treat older people susceptible to this condition.

**OBJECTIVES:**

To identify cutoff points for sociodemographic and anthropometric variables in screening probable and confirmed sarcopenia in community-dwelling older adults.

**DESIGN AND SETTING:**

This was a cross-sectional study of community-dwelling older adults in Araranguá, Santa Catarina, Brazil.

**METHODS:**

Sociodemographic (age, education) and anthropometric (weight, height, body mass index [BMI], waist circumference [WC], and dominant calf circumference [DCC]) factors were considered as predictors. The outcomes were probable sarcopenia (reduction in muscle strength assessed by time ≥ 15 s in the five-time sit-to-stand test) and confirmed sarcopenia (reduction in strength and muscle mass). Receiver operating characteristic curve analysis was used to analyze the ability to track sociodemographic and anthropometric variables for sarcopenia.

**RESULTS:**

In 308 older adults, WC > 91 cm in women and age > 69 years in men were useful in screening for probable sarcopenia. The variables age, weight, BMI, WC, and DCC can be used to screen for sarcopenia in older women and men.

**CONCLUSION:**

Sociodemographic and anthropometric variables are simple and accessible tools for sarcopenia screening in older adults.

## INTRODUCTION

Sarcopenia is a condition resulting from a reduction in muscle strength, mass, and performance.^1^ It is common in older adults and affects 10% of the older adult population worldwide,^2^ as well as 17% of Brazilian older adults.^3^ It is associated with negative health outcomes, such as increased mortality,^4^ risk of falls,^5^ functional disability,^6^ and prolonged hospitalization time.^7^

The European Working Group on Sarcopenia in the Elderly (EWGSOP2)^1^ proposed new diagnostic recommendations for early identification of this condition, in which the assessment should prioritize a reduction in muscle strength (classifying individuals with probable sarcopenia) using the five-time sit-to-stand test (5XSST) or handgrip strength (HGS) assessment.^1^ In addition to the reduction in muscle strength, it is also necessary to quantify the decrease in muscle mass, which should primarily be performed using computed tomography, magnetic resonance imaging, dual energy radiological absorptiometry (DXA), or bioimpedance analysis, to confirm the diagnosis.^1^ However, these assessments become unfeasible in clinical practice due to the high cost, risk of exposure to radiation, and low practicality.^1,8^

Underreporting of sarcopenia may occur in low- and middle-income countries that do not have easy access to these diagnostic tools, which cause the affected individuals to miss early intervention opportunities.^9^ Therefore, evidence has suggested the use of anthropometric markers, such as body mass index (BMI), waist circumference (WC), and dominant calf circumference (DCC), to track sarcopenia.^10,12^ Furthermore, Barbosa-Silva et al.^10^ observed an association between confirmed sarcopenia and the variables education level and age, without establishing cutoff points for these variables. Although sociodemographic variables such as age are nonmodifiable risk factors, access to cutoff points for screening sarcopenia can serve as a warning parameter for rehabilitation professionals. However, it is noteworthy that the diagnosis of sarcopenia in these studies was performed following the EWGSOP algorithm^13^ suggested in 2010, in which sarcopenia was identified by the reduction in muscle mass, unlike what has been updated and proposed by EWGSOP2, in which sarcopenia is initially diagnosed by a reduction in muscle strength. Thus, it is necessary to define the cutoff points of these indicators in screening probable and confirmed sarcopenia considering the new definitions proposed by EWGSOP2.^1^

Esteves et al.^9^ evaluated the use of anthropometric indicators in screening confirmed sarcopenia in older Brazilian adults using the EWGSOP2 algorithm.^1^ However, screening for probable sarcopenia and the use of sociodemographic variables were not considered. Tracking the disease in its early stage (probable sarcopenia) is extremely relevant in clinical practice, since the reduction in muscle strength can lead to difficulties in performing activities of daily living, such as sitting and standing up from a chair, balance, and walking.^1^

Thus, no cutoff points have been identified to date for age and other sociodemographic and anthropometric indicators in screening for sarcopenia in community-dwelling older adults using the EWGSOP2 algorithm.^1^

## OBJECTIVE

The aim of this study was to identify cutoff points in sociodemographic and anthropometric variables in screening probable and confirmed sarcopenia in community-dwelling older adults.

## METHODS

### Study design

This was a cross-sectional, household-based study with a probabilistic sample carried out in older adults from the municipality of Balneário Arroio do Silva, Santa Catarina, Brazil. Finite samples were calculated based on the total number of older adults registered (n = 2,833) in three basic health units (Unidade Básica de Saúde, UBS) of the city in 2018. An outcome prevalence of 50% was estimated with a five percentage point error (5 pp), and a 95% confidence interval (CI)^14^ for a total sample of 308 older adults. However, considering the possible sample losses, 540 older adults were eligible to be included in the sample.

### Population

Older adults were selected by drawing lots without replacement, considering the representative proportion of the total number of older adults registered in each UBS. Older people aged ≥ 60 years, who were residents of the community and able to perform 5XSST without the use of auxiliary devices were included in the study. Older adults who were bedridden and dependent, those who could not answer the questionnaires, residents in long-term care facilities, or those who had changed their residential addresses, were excluded. Losses were considered as older adults who were not found to be located at home after three attempts made on different days and times, and those who did not agree to participate in the study, and they were excluded. This study was approved by the Ethics Committee for Research with Human Beings of the Universidade Federal de Santa Catarina (UFSC) under the number CAAE no. 87776318.3.0000.0121 (dated June 22, 2018) and was conducted in accordance with the Declaration of Helsinki.

### Data collection procedure

The data were collected between September 2018 and September 2019. The selected older adults were initially contacted by telephone, and visits to their homes were scheduled. The team of interviewers was trained with the study instruments.

### Independent variables

The following sociodemographic variables were considered predictors: age (years) and education level (years of formal study), and anthropometric variables (body weight [kg], height [m], BMI, WC, and DCC).

During the assessment of body weight, older adults were instructed to wear a minimum amount of clothes and be barefoot. An anthropometric scale from the *Powner* brand was used with a capacity of up to 150 kg and a fraction of 100 g. Height was assessed after full inspiration with the spine supported on the wall, bare feet, and aligned.^15^ Weight and height were considered for the assessment of BMI, which was obtained with the calculation suggested by the World Health Organization: “weight/height².”^16^

A *Cescorf* brand inelastic tape was used to assess WC and DCC. WC was measured by marking the midpoint between the lower edge of the last rib and the upper edge of the iliac crest. For standardization purposes, DCC was measured with the older adults standing with their feet 20 cm apart in the region of maximum circumference in the plane perpendicular to the longitudinal line of the calf.^15^

### Study outcomes

Probable and confirmed sarcopenia were considered as the study outcomes. The assessment of probable sarcopenia was performed using 5XSST, which measured the time taken to sit and stand up from a chair in five repetitions, with arms crossed over the chest.^17^ Older adults who spent more than 15 s in the test were classified as probable sarcopenic.^1,18^

In addition to a reduction in muscle strength, older adults should also show a reduction in muscle mass to confirm sarcopenia.^1^ Thus, the equation proposed by Lee et al.,^19^ validated for use in older Brazilian adults,^20^ was used to assess the reduction in muscle mass. It presented a high correlation rate in the community-dwelling older adult population (r = 0.86 for women and r = 90 for men), in addition to high specificity (89%) and sensitivity (86%) when compared with the DXA method.^20^

Lee's Equation: SM(kg)=(0.244*BW)+(7.8*Ht)+(6.6*gender)−(0.098*age)+(race−3.3)

where SM: skeletal muscle; BW: body weight (kg); Ht: height (m); gender: 1 for male and 0 for female; race: −1.2 for Asian, 1.4 for African American, and 0 for Caucasian or Hispanic.

After defining the skeletal muscle mass, the adjustment for height squared was performed, and the muscle mass index (MMI) was obtained.^20^ The cutoff point used to identify muscle mass loss was the lowest 20% percentile of the population distribution.^21^ In this study, MMI values < 6.700 kg/m^2^ in women and < 9.60 kg/m^2^ in men were considered confirmed sarcopenia, similar to the data found in the literature.^9^

### Adjustment variables

After defining the cutoff points in screening sarcopenia, multivariate logistic regression analyses were performed to verify the association between the variables, considering the following adjustment variables: multimorbidity (concurrent presence of two or more self-reported chronic diseases),^22^ depressive symptoms (a score ≥ 5 on the Geriatric Depression Scale),^23^ level of leisure-time physical activity assessed by the International Physical Activity Questionnaire validated in Brazil^24,25^ (categorized as sufficiently active [> 150 min] and insufficiently active [< 150 min])^26–28^ and history of falls in the last 12 months.^29^

### Data analysis

Data were collected and independently checked by two researchers and entered into the SPSS database (IBM, Chicago, Illinois, United States), version 23.0. The significance level adopted was 5%. Categorical variables were described using absolute and relative frequencies and their respective 95% CIs.

A receiver operating characteristic curve was constructed using the MedCalc software (MedCalc Software, Ostende, Belgium) version 19.1 to assess the ability to track sociodemographic and anthropometric variables for probable and confirmed sarcopenia. Multivariate logistic regression analyses were performed to assess the associations between variables and estimate the crude and adjusted odds ratios with 95% CIs.

## RESULTS

**Figure 1:**
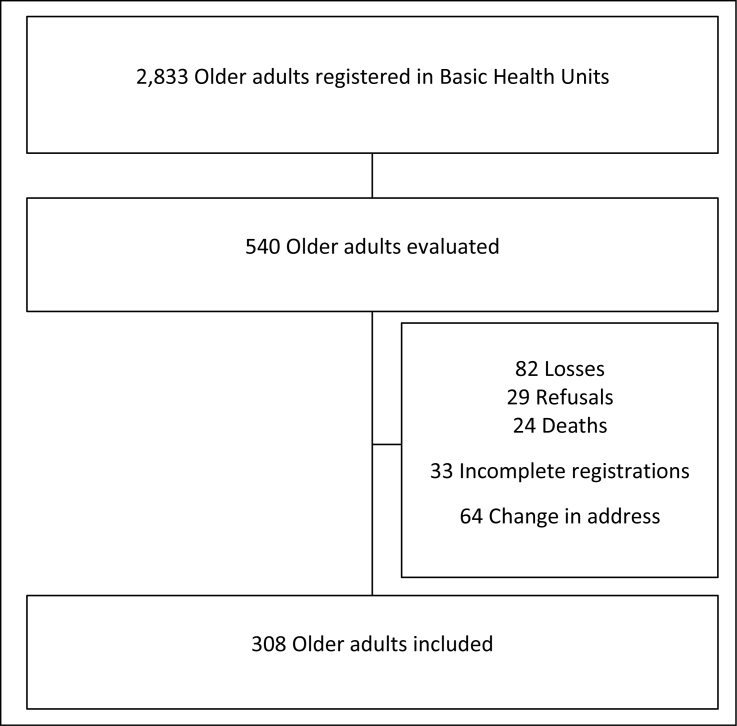
Flowchart depicting the sample selection process.

Among the 540 eligible older adults, 64 were excluded from the study due to a change in address, 33 due to incomplete registrations, 29 due to refusal to participate, and 24 due to death, along with 82 losses, totaling 308 older adults evaluated in the study (Figure 1).

The sample consisted of 57.80% (178) older women, with a mean age of 69.91 ± 7.31 years and with 5.40 ± 3.64 years of schooling. Males accounted for 42.20% (130) of the sample, with a mean age of 69.80 ± 6.71 years and with 6.07 ± 3.83 years of formal education. The prevalence of probable sarcopenia was 50.60% in women and 38.30% in men, and that of confirmed sarcopenia was 6.80% in women and 8.10% in men. Sociodemographic and anthropometric variables of the participants are presented in Table 1.

**Table 1:** Sociodemographic and anthropometric characteristics of the sample (n = 308)

Characteristic (mean ± SD)			Probable Sarcopenia		Confirmed Sarcopenia	
			No Sarcopenia	Sarcopenia	No Sarcopenia	Sarcopenia
**Female (n, %)**			78 (49.40%)	80 (50.60%)	151 (93.20%)	11 (6.80%)
	***Sociodemographic***					
		Age (years)	68.43 ± 6.10	69.8 ± 7.42	68.68 ± 6.18	78.18 ± 10.81*
		Education (years)	6.05 ± 3.67	4.93 ± 3.73*	5.64 ± 3.78	3.72 ± 2.64
	***Anthropometric***					
		Body weight (kg)	69.11 ± 13.94	73.02 ± 14.98	73.58 ± 15.17	55.00 ± 4.28*
		Height (m)	1.54 ± 0.06	1.55 ± 0.06	1.55 ± 0.06	1.54 ± 0.06
		BMI (kg/m²)	28.98 ± 5.67	30.04 ± 5.81	30.47 ± 5.98	23.29 ± 2.84*
		WC (cm)	96.26 ± 11.79	100.51 ± 11.10*	99.88 ± 12.06	89.72 ± 6.23*
		DCC (cm)	37.68 ± 4.17	37.74 ± 3.84	38.61 ± 5.13	33.18 ± 1.32*
**Male (n, %)**			74 (61.70%)	46 (38.30%)	113 (91.90%)	10 (8.10%)
	***Sociodemographic***					
		Age (years)	68.82 ± 6.67	71.45 ± 6. 55*	69.33 ± 6.59	74.30 ± 6.39*
		Education (years)	6.28 ± 3.39	5.32 ± 3.91	6.01 ± 3.49	5.00 ± 4.89
	**Anthropometric**					
		Body weight (kg)	79.78 ± 17.07	78.63 ± 15.35	80.58 ± 16.14	64.90 ± 9.64*
		Height (m)	168.52 ± 6.54	166.86 ± 7.21	167.57 ± 6.58	170.70 ± 8.65
		BMI (kg/m²)	28.02 ± 5.49	28.19 ± 5.04	28.63 ± 5.15	22.13 ± 1.69*
		WC (cm)	102.37 ± 13.29	105.47 ± 14.15	104.69 ± 13.45	91.45 ± 8.26*
		DCC (cm)	37.18 ± 4.00	36.83 ± 5.78	37.36 ± 4.77	33.39 ± 1.61*

* Differences between groups with and without sarcopenia (P < 0.05).

SD = standard deviation; BMI = body mass index; WC = waist circumference; DCC = dominant calf circumference.

The variable capable of tracking probable sarcopenia in older women was WC, with a cutoff point of > 91 cm. Age, formal education, weight, height, BMI, and DCC in women had no screening ability for probable sarcopenia (Table 2). The predictor variable for screening for probable sarcopenia in men was age, with a cutoff point of > 69 years. However, formal education, weight, height, BMI, WC, and DCC had no significant ability to track probable sarcopenia (Table 2).

**Table 2:** Accuracy of the anthropometric and sociodemographic variables for screening probable sarcopenia (n = 308)

Variable			Predictive value	AUC (CI 95%)	Sensitivity (CI 95%)	Specificity (CI 95%)	+LR (CI 95%)	−LR (CI 95%)
**Female (n = 178)**								
	***Sociodemographic***							
		Age (years)	--	0.54 (0.45; 0.63)	--	--	--	--
		Education (years)	--	0.59 (0.50; 0.68)	--	--	--	--
	***Anthropometric***							
		Body weight (kg)	--	0.57 (0.48; 0.66)	--	--	--	--
		Height (m)	--	0.55 (0.47; 0.64)	--	--	--	--
		BMI (kg/m²)	--	0.55 (0.46; 0.64)	--	--	--	--
		WC (cm)	> 91	0.61 (0.53; 0.69)*	82.50% (72.4; 90.1)	42.31% (31.2; 54.0)	1.43 (1.2; 1.8)	0.41 (0.2; 0.7)
		DCC (cm)	--	0.51 (0.42; 0.60)	--	--	--	--
**Male (n = 130)**								
	***Sociodemographic***							
		Age (years)	> 69	0.62 (0.52; 0.70)*	65.22% (49.8; 78.6)	60.27% (48.1; 71.5)	1.64 (1.2; 2.3)	0.58 (0.4; 0.9)
		Education (years)	--	0.60 (0.49; 0.70)	--	--	--	--
	***Anthropometric***							
		Body weight (kg)	--	0.53 (0.42; 0.64)	--	--	--	--
		Height (m)	--	0.56 (0.45; 0.67)	--	--	--	--
		BMI (kg/m²)	--	0.51 (0.40; 0.62)	--	--	--	--
		WC (cm)	--	0.55 (0.44; 0.66)	--	--	--	--
		DCC (cm)	--	0.50 (0.39; 0.61)	--	--	--	--

* P < 0.05;

AUC = area under the ROC curve; ROC = receiver operating characteristic curve; +LR: odds ratio for positive test; −LR: odds ratio for negative test.

BMI = body mass index; WC = waist circumference; DCC = dominant calf circumference; CI = confidence interval.

For confirmed sarcopenia in women, the analysis showed that age (> 76 years), weight (≤ 58 kg), BMI (≤ 27.66 kg/m²), WC (≤ 92 cm) and DCC (≤ 35 cm) were able to track confirmed sarcopenia. Tracking ability was not observed for education and height. In men, age (> 73 years), weight (≤ 71 kg), BMI (≤ 24.45 kg/m²), WC (≤ 98 cm) and DCC (≤ 34 cm) were able to track confirmed sarcopenia. Education level and height were not able to track confirmed sarcopenia in men (Table 3).

**Table 3:** Accuracy of the anthropometric and sociodemographic variables for screening confirmed sarcopenia (n = 308)

Variable			Predictive value	AUC (CI 95%)	Sensitivity (CI 95%)	Specificity (CI 95%)	+LR (CI 95%)	−LR (CI 95%)
**Female (n = 178)**								
	***Sociodemographic***							
		Age (years)	> 76	0.75 (0.68; 0.82)*	72.73% (39.0; 94.0)	86.75% (80.3; 91.7)	5.49 (3.2; 9.5)	0.31 (0.1; 0.8)
		Education (years)	--	0.64(0.49; 0.79)	--	--	--	--
	***Anthropometric***							
		Body weight (kg)	≤ 58	0.90 (0.85; 0.94)*	90.91% (58.7; 99.8)	87.42% (81.0; 92.3)	7.22 (4.6; 11.4)	0.10 (0.02; 0.7)
		Height (m)	--	0.52 (0.35; 0.69)	--	--	--	--
		BMI (kg/m²)	≤ 27.66	0.88 (0.82; 0.93)*	100.00% (71.5; 100.0)	66.89% (58.8; 74.3)	3.02 (2.4; 3.8)	0.00
		WC (cm)	≤ 92	0.76 (0.69; 0.83)*	81.82% (48.2; 97.7)	72.67% (64.8; 79.6)	2.99 (2.0; 4.4)	0.25 (0.07; 0.9)
		DCC (cm)	≤ 35	0.88 (0.82; 0.93)*	100.00% (71.5; 100.0)	78.52% (71.1; 84.8)	4.66 (3.4; 6.3)	0.00
**Male (n = 130)**								
	***Sociodemographic***							
		Age (years)	> 73	0.71 (0.62; 0.79)*	60.00% (26.2; 87.8)	74.11% (65.0; 81.9)	2.32 (1.3; 4.2)	0.54 (0.3; 1.2)
		Education (years)	--	0.62 (0.42; 0.82)	--	--	--	--
	***Anthropometric***							
		Body weight (kg)	≤ 71	0.81 (0.73; 0.88)*	90.00% (55.5; 99.7)	73.21% (64.0; 81.1)	3.36 (2.3; 4.9)	0.14 (0.02; 0.9)
		Height (m)	--	0.66 (0.48; 0.83)	--	--	--	--
		BMI (kg/m²)	≤ 24.45	0.92 (0.85; 0.96)*	100.00% (69.2; 100.0)	83.93% (75.8; 90.2)	6.22 (4.1; 9.5)	0.00
		WC (cm)	≤ 98	0.82 (0.74; 0.88)*	90.00% (55.5; 99.7)	72.97% (63.7; 81.0)	3.33 (2.3; 4.8)	0.14 (0.02; 0.9)
		DCC (cm)	≤ 34	0.85 (0.77; 0.91)*	80.00% (44.4; 97.5)	85.71% (77.8;91.6)	5.60 (3.2; 9.7)	0.23 (0.07; 0.8)

* P < 0.05;

AUC: Area under the ROC curve; ROC: receiver operating characteristic curve; +LR: odds ratio for positive test; −LR: odds ratio for negative test.

BMI = body mass index; WC = waist circumference; DCC = and dominant calf circumference; CI = confidence interval.

In the adjusted multivariate logistic regression analysis, older women with WC > 91 cm had a 3.05 (95% CI: 1.40; 6.61) times greater chance of having probable sarcopenia than older women with WC < 91 cm. Older adults aged > 69 years were 2.56 (95% CI: 1.12; 5.82) times more likely to have probable sarcopenia than those aged < 69 years (Table 4).

**Table 4:** Results of multivariate logistic regression analysis between predictor variables and probable sarcopenia in community-dwelling older adults (n = 308)

Variables		Probable Sarcopenia	
		Unadjusted OR (CI 95%)	Adjusted^a^ OR (CI 95%)
***Female (178)***			
**WC > 91 cm**			
	No	1.00	1.00
	Yes	3.41 (1.61; 7.24)	**3.05 (1.40; 6.61)** *
***Male (130)***			
**Age > 69 years**			
	No	1.00	1.00
	Yes	2.55 (1.15; 5.59)	**2.56 (1.12; 5.82)** *

a Adjusted for multimorbidity, depressive symptoms, level of leisure-time physical activity, and history of falls;

* P < 0.05.

WC = waist circumference; OR = odds ratio; CI = confidence interval.

Due to the low prevalence of sarcopenia confirmed in the sample, performing a multivariate logistic regression analysis for this condition was not possible.

## DISCUSSION

The data from this study showed that WC > 91 cm in women and age > 69 years in men should be used in screening for probable sarcopenia. Age, weight, BMI, WC, and DCC were screening variables for both women and men for confirmed sarcopenia.

The prevalence of probable sarcopenia and confirmed sarcopenia in the present study was 50.60% and 6.80% in women and 38.30% and 8.10% in men, respectively. The prevalence of probable sarcopenia observed in this study was higher than that found by Wearing et al.^30^ who reported it to be 26.3% for women and 28.0% for men in community-dwelling older Swiss adults. This difference in the reported prevalence of probable sarcopenia may be related to the sociodemographic, ethnic, and economic characteristics of the samples, as well as the measurement method, since probable sarcopenia was evaluated using 5XSST in this study, whereas in the study by Wearing et al.,^30^ HGS was used.

Regarding the prevalence of confirmed sarcopenia, 6.80% of the women and 8.10% of the men had this condition. Similar findings were obtained by Esteves et al.^9^ who observed a prevalence of 6.10% of confirmed sarcopenia in older Brazilian adults. Moreover, confirmation of sarcopenia was obtained with a reduction in muscle mass as assessed by Lee's equation^19^ in the same manner as in the present study. These findings show the difference in prevalence when considering probable and confirmed sarcopenia, making it necessary to measure strength and muscle mass in older adults in clinical practice for early detection of the disease to reduce underreporting of sarcopenia in this population.

The present study suggests that an age > 69 years may be indicative of probable sarcopenia in men. Fragala et al.^18^ observed that, in men, as muscle quality decreased, the time taken to perform 5XSST increased. Bai et al.^31^ demonstrated that reduction in muscle strength directly affects the physical performance of older adults with aging. In addition, the literature shows that type II muscle fibers suffer neurodegeneration with aging, causing muscle tissue impairment, confirming the association between sarcopenia and age.^32^ It is known that age is also related to confirmed sarcopenia, with higher prevalence in older age groups.^10,33^ Data from the present study suggest that age > 73 years is a good determinant in screening for confirmed sarcopenia in men. Although age has shown significant results in screening probable and confirmed sarcopenia, no other study to date has suggested cutoff points for this variable.

Confirmed sarcopenia was screened in women aged > 76 years. This finding corroborates that of Albani et al.,^34^ who observed a decrease in the concentration of growth factors similar to insulin type 1 in women aged 70 years. This growth factor is responsible for muscle growth and repair and is a triggering factor for the development of sarcopenia in older women.^33^ Despite age being a nonmodifiable risk factor, the identification of cutoff points enables a warning sign for rehabilitation professionals, resulting in early diagnosis and intervention for the disease.

WC > 91 cm in women stands out as a possible anthropometric indicator for screening for probable sarcopenia. Evidence indicates that the accumulation of visceral fat in women may have a multifactorial cause, involving lifestyle, hormonal factors, body composition, reduced synthesis, and innervation of muscle proteins, in addition to impaired intramyocellular calcium metabolism.^18,35,36^ In addition, the accumulation of visceral fat reduces muscle quality due to fat infiltration in the tissue, affecting muscle strength. Consequently, it can affect the functional capacity of older adults in aggravated circumstances, causing an excess of fat mass associated with a reduction in strength, termed sarcopenic obesity.^37^ Kim et al.^38^ observed that high WC (88.4 ± 9.1) was positively associated with functional limitation in older women, reinforcing the findings of this study. Furthermore, it appears that concomitant with the increase in WC, elevations in the levels of proinflammatory cytokines, such as tumor necrosis factor α, interleukin (IL)-6, and IL-1, are observed.^39^ These act directly on skeletal muscle to facilitate muscle catabolism through pathways related to chronic inflammation and oxidative stress, thus contributing to the development of sarcopenia.^35,39^

Considering WC as a screening parameter for confirmed sarcopenia, cutoff points ≤ 92 and ≤ 98 cm are suggested for women and men, respectively. Baker et al.^40^ observed that high adiponectin concentrations are associated with weight loss, low density, and skeletal muscle mass, in addition to functional limitation in older adults aged 70–79 years, which may be a factor for the development of sarcopenia in individuals of this age group.^41^ Casals et al.^42^ observed that the reduction of muscle mass in older adults can negatively affect glucose regulation, impacting muscle tissue. When comparing the results of this study with those of Esteves et al.^9^ (WC: ≤ 86 cm for women and ≤ 97 cm for men) and Confortini et al.^11^ (WC: 88 cm for women and 92 cm for men), the results were higher in sensitivity for both sexes and in specificity for men, reinforcing the utility of WC as a viable indicator for sarcopenia in clinical practice.

Weight and BMI proved to be effective variables for screening for confirmed sarcopenia in both sexes. The cutoff points for weight were ≤ 58 and ≤ 71 kg for women and men, respectively. For BMI, the suggested values were ≤ 27.66 kg/m² for women and ≤ 24.45 kg/m² for men. Beaudart et al.^8^ observed a strong association between BMI and muscle mass reduction in older adults with sarcopenia. Although BMI is not only related to muscle mass, it is believed that lower values in older people with sarcopenia are due to disease. characteristics, such as reduced muscle mass.^9,11,12^ Studies using BMI as a predictor for confirmed sarcopenia found results similar to those of this study, suggesting a cutoff point for women at ≤ 24.5 kg/m² (sensitivity: 100.00%; specificity: 81.78%) and for men at ≤ 24.8 kg/m² (sensitivity: 100%; specificity: 74.22%).^9^ BMI cutoff values were also suggested by Confortin et al.^11^ for women at 26.2 kg/m² (sensitivity: 74.60%; specificity: 85.70%) and for men at 24.6 kg/m² (sensitivity: 84.90%; specificity: 63.30%), confirming BMI as a good indicator for screening for sarcopenia.

Based on the data analyzed, DCC could also be used as an anthropometric variable to predict confirmed sarcopenia in both sexes. Studies using DCC to predict confirmed sarcopenia observed similar results to those found in the current study. For example, Barbosa-Silva et al.^10^ suggested a cutoff point of ≤ 33 cm (sensitivity: 100.00%; specificity: 76.00%) for women and ≤ 34 cm (sensitivity: 61.00%; specificity: 76.00%) for men. Esteves et al.^9^ also suggested DCC cutoff points of ≤ 31 cm (sensitivity: 93.33%; specificity: 67.05%) for women and ≤ 33 cm (sensitivity: 90.00%; specificity: 60.16%) for men. In addition, the study translating SARC-F questionnaire into Portuguese (Brazilian) proposed that using the instrument, DCC should be measured in Brazilian older adult population with a cutoff point of ≤ 33 cm for women and ≤ 34 cm for men, with lower sensitivity and specificity than those found in this study.^43^

These findings suggest the utility of DCC in screening for confirmed sarcopenia.^9,11,12,43^ DCC is a sensitive anthropometric measure for muscle mass in older adults.^44^ This is a useful factor to detect the presence of confirmed sarcopenia when there is a reduction in muscle mass in this population. However, the use of DCC in older adults has limitations, such as the impossibility of separating muscle tissue from intramuscular or subcutaneous adipose tissue.^43^ The use of DCC is unfeasible in the detection of probable sarcopenia, as its diagnosis will only be made when a reduction in muscle strength is observed.

Thus, as in the findings of this study, WC, DCC, and BMI are shown as good indicators in the literature in screening for sarcopenia in the older adult population in general.^9–11^ However, the use of gold standard instruments is recommended to assess muscle mass in obese individuals, as the values will be far below the suggested cutoff points due to possible sarcopenic obesity characterized by dysregulated secretion of adipokines, proinflammatory cytokines, and decreased adiponectin, which cause expansion and dysfunction in the adipose tissue. This, in turn, induces catabolism, chronic inflammation, and increased secretion of proinflammatory myokines in the muscle tissue, causing muscle dysfunction and exacerbation of adipose tissue inflammation, thus establishing a vicious cycle triggering the pathogenic cascade of the disease.^39,43,45^

Despite the relevance of the findings, some limitations should be highlighted, such as the use of the Lee equation to measure muscle mass. Although the use of this equation demonstrates a high correlation rate in community-dwelling older adult population when compared with DXA, it is not considered the gold standard for muscle strength assessment. However, considering the practical applicability of these findings, these diagnostic tools are not easily available, in addition to exposing older adults to high levels of radiation. Furthermore, it is noteworthy that it was not possible to perform multivariate logistic regression analysis for the confirmed sarcopenia sample because of the small sample size for this category (n = 21).

On the other hand, the study's strong point is the recommendation of sociodemographic and anthropometric cutoff points that help in the screening for early stage sarcopenia and confirming the condition in community-dwelling older adults. In addition, it is highlighted that this screening can be carried out through low-cost, easy, and quick assessments, enabling health professionals to carry out early and effective interventions for the disease in clinical practice.

## CONCLUSION

Sociodemographic and anthropometric variables are simple and accessible tools in screening for sarcopenia in older people. In this sense, our data suggest the use of waist circumference for women and age for men as variables capable of tracking probable sarcopenia in older adults.
